# MRI radiomic signature predicts peritumoral brain edema resolution following meningioma surgery

**DOI:** 10.1007/s00701-025-06746-7

**Published:** 2025-12-03

**Authors:** Sergio Garcia-Garcia, Joonas Laajava, Juuso Takala, Mika Niemelä, Miikka Korja

**Affiliations:** Department of Neurosurgery, Helsinki University Hospital, Haartmaninkatu 4, 00290 Helsinki, Finland

**Keywords:** Radiomics, Machine learning, Meningioma, Edema, Peritumoral, Magnetic resonance

## Abstract

**Background:**

Intracranial meningiomas(IM) are often associated with peritumoral brain edema(PTBE), visible as hyperintensities on T2/FLAIR MRI. Postoperative persisting PTBE-like changes likely represent gliosis that, in turn, contributes to surgical morbidity. Since the human eye is unable to distinguish between PTBE and gliosis on MR images, we hypothesized that radiomic analysis of preoperative peritumoral T2/FLAIR hyperintensities could distinguish preoperatively established gliosis from reversible edema.

**Methods:**

MRI of patients with gross totally resected IM were retrospectively analyzed. Preoperative and 1-year postoperative PTBE were segmented on MRI. One-year MRI were classified into two categories based on whether PTBE resolution exceeded 80% of the initial volume. RF were extracted from meningioma and PTBE regions on T1-contrast-enhanced, T2, and FLAIR MRI sequences. The dataset was split into training, validation, and test cohorts(70–10-20%). Feature reduction used correlation-based exclusion and recursive feature elimination with cross-validation. Nine ML algorithms were trained and evaluated, and best model’s interpretability assessed using Shapley Additive Explanations(SHAP).

**Results:**

644 RF were extracted per individual from the pre and postoperative MRI of 123 operated patients. The Random Forest model utilizing 10 RF achieved the best performance (accuracy:0.91;precision:0.92;F1-score:0.92;ROC-AUC:0.94), demonstrating radiomics’ utility in predicting PTBE resolution at 1-year post-surgery. SHAP analysis provided interpretability, highlighting key RF, differences between patient groups, and potential sources of algorithmic error.

**Conclusions:**

These results underscore the potential of radiomics and ML to accurately predict postoperative PTBE resolution, differentiating transient PTBE from persistent PTBE-like changes (gliosis). This study provides initial insights into the potential of advanced imaging and computational techniques for non-invasive preoperative assessment, which may contribute to more personalized surgical strategies.

**Supplementary Information:**

The online version contains supplementary material available at 10.1007/s00701-025-06746-7.

## Introduction

Intracranial meningiomas(IMs) are the most frequently diagnosed primary intracranial tumors, accounting for over one-third of all cases [27]. Moreover, meningiomas are the most frequent incidentally diagnosed intracranial neoplasm [16]. Their incidence increases with age and shows a female predominance (2:1) [27]. IMs frequently exhibit slow growth and low invasiveness, leading to their classification as benign tumors. However, these tumors can still cause significant morbidity manifesting as focal neurological deficits, seizures, and reduced quality of life.

Surgical series of IMs report peritumoral brain edema(PTBE) in 38–67% of cases [13]. This PTBE is commonly defined as hyperintense areas on T2-weighted magnetic resonance imaging(MRI) or fluid-attenuated inversion recovery (FLAIR) sequences [13]. PTBE is more frequent in brain-invasive and secretory subtypes of meningiomas, older patients and bigger tumors [24]. Preoperative PTBE is also linked to higher recurrence rates, postoperative hemorrhages and increased risk of seizures [2, 8, 34]. Furthermore, PTBE correlates with longer hospital stays, postoperative dependence, and increased mortality [24, 32, 33]. While PTBE has been related to factors such as tumor volume, location, venous outflow obstruction, pial supply and, more recently, to glymphatic dysfunction, its pathogenesis remains unclear [19, 30, 34, 37, 38, 40].

A recent review on PTBE suggested that some preoperative T2/FLAIR hyperintensity may represent gliosis rather than edema [21]. Indeed, in our series, some PTBE-like changes, likely gliosis, persisted in 97% of gross totally resected IMs, while over 90% PTBE volume resolution was observed in about one-third of these patients after surgery [22]. Unfortunately, PTBE and gliosis appear similar on MRI and cannot be visually distinguished. This uncertainty arises because a considerable proportion of the hyperintensity observed preoperatively as PTBE on T2/FLAIR in fact represents irreversible gliosis rather than resolvable edema. This underscores the need for innovative methodological approaches capable of differentiating reversible edema from permanent gliotic changes that are already present in preoperative MRI, thereby facilitating accurate preoperative characterization of gliosis and a more reliable prediction of postoperative neurological recovery.

Radiomics, a rapidly advancing field, involves the extraction of high-dimensional quantitative features from medical images [11, 23]. This allows for a more comprehensive evaluation of radiological images than traditional visual assessment [1]. Radiomic analyses generate a vast number of features necessitating the use of advanced statistical methods to distill this data into meaningful insights [4]. Machine learning(ML), a subset of artificial intelligence, plays a crucial role in managing these large datasets, identifying the most relevant features, and training classification models to produce refined and explainable results [4].

In this study, we aimed to assess the radiomic characteristics of tumoral and peritumoral T2/FLAIR hyperintensity (HI) in patients undergoing surgery for IMs to develop a preliminary classification model. We hypothesized that the presented radiomics and ML approach could accurately predict PTBE resolution after meningioma surgery.

## Methods

This research follows CLEAR guidelines (Suplementary Table 1) [20]. The local institutional review board at Helsinki University Hospital approved the study design and retrospective collection of data and images. In compliance with local data privacy laws, all patient´s images and clinical information were processed and analyzed in a cybersecure operating environment (Blinded for review).

**Table 1:** Summary of sample characteristics

Variable	Distribution	Mean/Median (SD/IQR)	Mean/Median (SD/IQR)		Test	Mean Difference (95%CI) Effect Size (95%CI)	*p*-value
			Resolution				
		Total	< 80%	> 80%			
Number		123	48	75			
Sex: F/M		79/44	30/18	49/26	X^2^ = 0.001		0.974
Age	N	57.2 (13.1)	59.9 (12.9)	55.6(13.1)	t = 1.75	4.3 (−0.56–9.24)	0.08
WHO grade 1/2		80/43	38/10	42/33	X^2^ = 5.12		0.023
Ed. Vol. cm3(T2/FLAIR)	No-N	23.1 (40.8)	18.1 (30.0)	28.3(50.3)	U-MW = 1329.0	−9392(−147,207- 127,468)	0.015
Tumor Vol. cm^3^ (T1CE)	No-N	20.9(40.8)	28.9(52.9)	20.3 (35.1)	U-MW = 2061.0	5130(−77,970- 84,191)	0.18
TSA cm^2^ (T1CE)	No-N	43.5 (55.0)	57.2(57.3)	42.2 (50.0)	U-MW = 2076.0	844(−9126- 9884)	0.15
Resolution of Ed	No-N	87.2% (25.6)	62.3% (7.9)	94.8% (7.9)			

Variables with normal(N) distribution are described by Mean and Standard Deviation (SD). Variables with no normal (No-N) distribution are expressed by median and Interquartile range (IQR)instead. Parametric (Mean Difference) and no parametric (Effect Size) statistical tests are performed to assess differences in the distribution of variables across groups defined as higher or lower to 80% resolution of the edema. *CI* Confidence Interval, *Ed* Edema, *F* Femal, *M* Male, *t* Student’s t test, *T1CE* T1 contrast enhanced, *TSA* Tumor Surface Area, *Vol* Volume, *U-MW* Mann–Whitney U test, *X*^*2*^ Chi-squared Test

### Patient selection

**Fig. 1:**
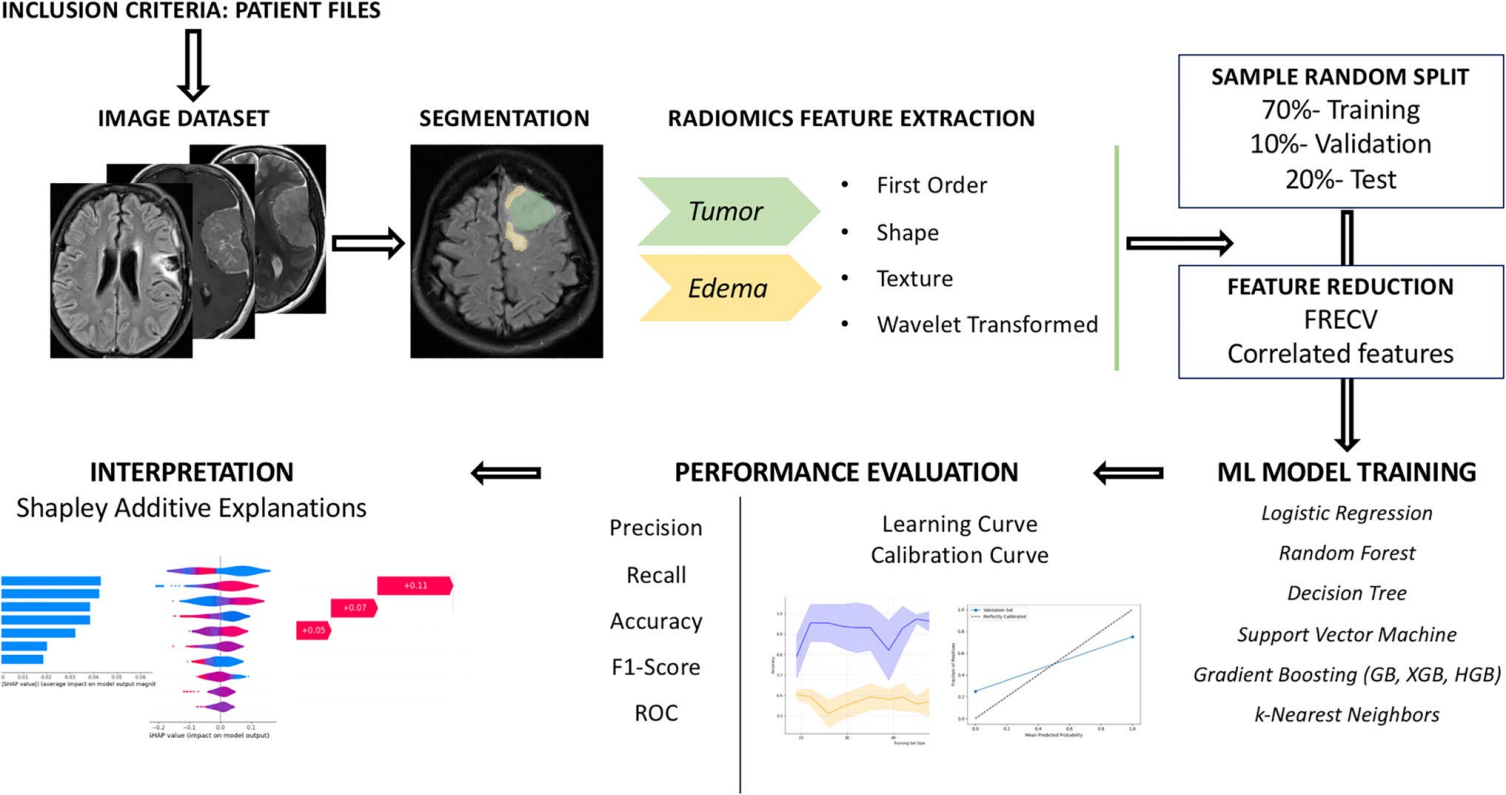
Diagram depicting an overview of the research methodology

Adult patients with intracranial convexity, parasagittal or falcine meningiomas who underwent gross total resection (GTR) were retrospectively identified and included in this study. Eligibility criteria required the availability of high-quality preoperative and one-year follow-up MRI scans with T1-weighted contrast-enhanced (T1CE), T2, and FLAIR sequences. Only patients demonstrating at least partial postoperative reduction in PTBE on follow-up imaging were included in the analysis. Patients with an increase in PTBE postoperatively—suggestive of iatrogenic injury—were excluded (Fig. 1).

### Imaging acquisition

MRI studies were obtained using standard clinical protocols [22]. Files were converted and stored for further processing as Neuroimaging Informatics Technology Initiative (NIfTI) files. Further details regarding imaging protocols and the study population this research is partially based on can be found in our previous publication [22].

### Image segmentation

**Fig. 2:**
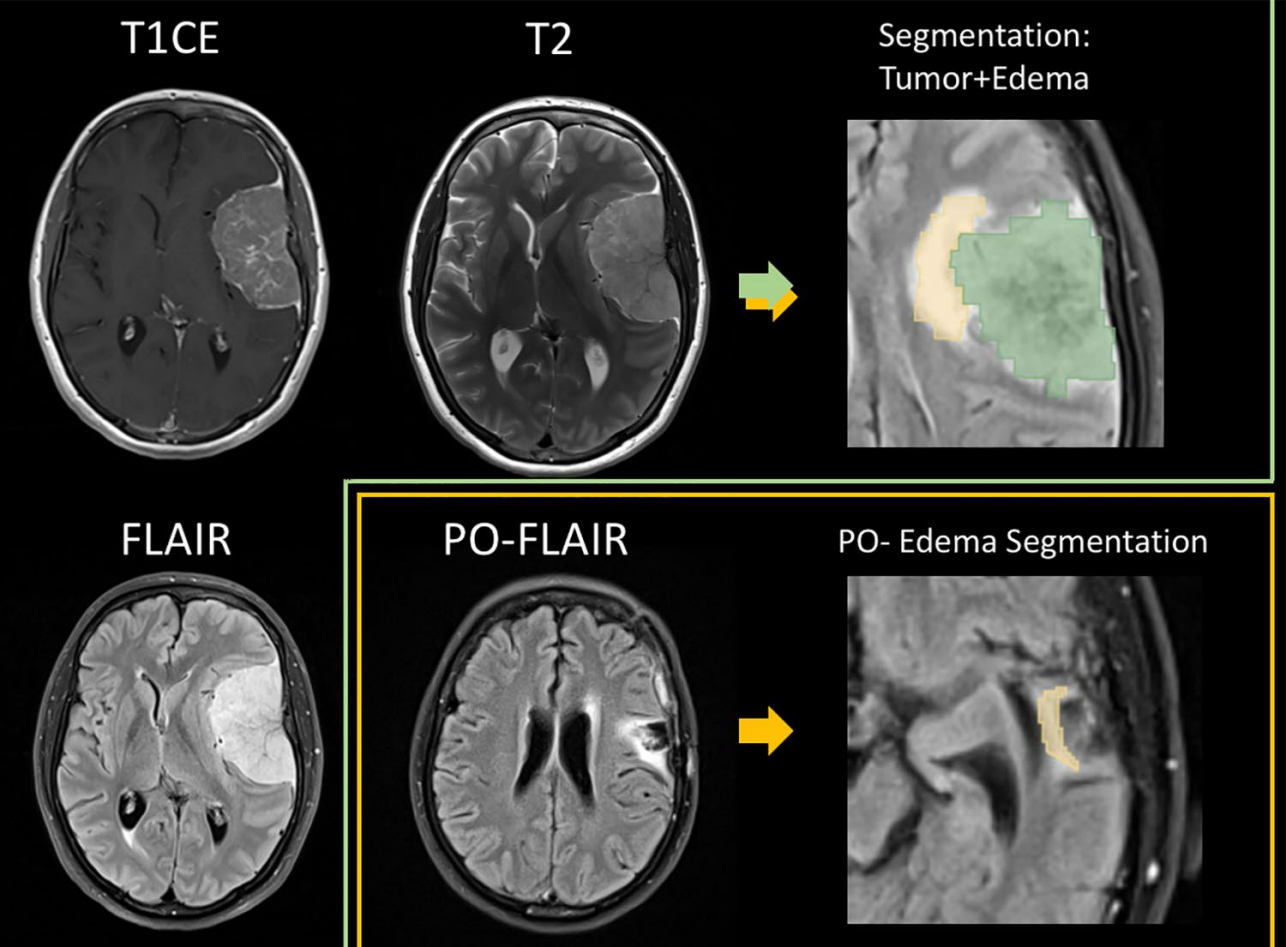
Axial views of MRI sequences T1 Contrast enhanced (T1CE); T2 and FLAIR of one of the cases included in the study illustrating the segmentation process. Tumor segmentation is shadowed in green while pre and postoperative(PO) edema segmentation is shadowed in yellow

Tumor segmentation was performed on preoperative T1CE images using 3D Slicer (v.5.6, https://www.slicer.org/) [18]. PTBE was segmented on preoperative and one-year follow-up FLAIR sequences. Semi-automatic segmentation was performed by a trained operator and reviewed by a neurosurgeon with more than 10 years of experience in neuroimaging to ensure accuracy and consistency (Fig. 2).

### Edema volume and resolution

PTBE volume was calculated from segmented regions on FLAIR sequences. Edema resolution at one year was quantified as the change in edema volume between preoperative and 1-year postoperative FLAIR. The resolution was expressed as percentage, and the resolution percentage was dichotomized using a cut-off value of 80% to classify edema as persistent or substantial decrease. The threshold of 80% was selected to ensure adequate representation within both categories while accounting for a clinically significant resolution.

### Software and computational resources

Python (version 3.10) was used for programming. Libraries included SimpleITK, NumPy, Pandas, PyRadiomics, sklearn, xgboost and shap. Matplotlib was used for graphics. All computations were performed in Microsoft Azure Machine Learning using a compute instance(CPU type, 14 GB RAM) running Linux Ubuntu 22.04.05 operating system.

### Image co-registration

To ensure spatial alignment across the MR sequences, the T2 and FLAIR sequences from each patient were co-registered to T1CE using the SimpleITK-SimpleElastix library.

### Radiomic feature extraction

Intensity normalization was performed. Radiomic features were extracted from tumor and preoperative edema segmentations of each sequence using PyRadiomics library in default mode, which includes IBSI (Image Biomarker Standardization Initiative)-compliant feature calculations and optimized C-based extensions for enhanced computational efficiency. Regarding quantization, different binWidth (bW) configurations were used (5, 10, 15, 20, 25, and 30). This parameter controls the discretization of intensity values for texture analysis. Testing different bW allowed assessment of robustness by identifying features that remained stable across varying quantization levels, determining the optimal setting for extracting the most informative features, and evaluating how discretization impacts feature performance in ML models.

The extractor was configured to compute the default set of radiomic feature encompassing First order, Shape, Texture and Wavelet-Transformed features. Extracted Radiomic Features encompassed the following categories:

- First-Order: Features that describe the distribution of voxel intensities within the segmented labels without considering spatial relationships (i.e. mean, median, standard deviation, variance, skewness, kurtosis, energy, entropy).
- Shape: Quantitative measures of the geometric properties of the segmentation, independent of intensity values (volume, surface area, sphericity, compactness, maximum 3D diameter, elongation, flatness)
- Texture: Higher-order statistics that capture spatial patterns and relationships of voxel intensities within the segmentation, derived from various matrices: Gray-Level Co-Occurrence Matrix (GLCM)(Contrast, correlation, energy, homogeneity, dissimilarity, entropy); Gray-Level Run Length Matrix (GLRLM) (Short run emphasis, long run emphasis, gray-level non-uniformity, run length non-uniformity, run percentage, run entropy); Gray-Level Size Zone Matrix (GLSZM)(Small area emphasis, large area emphasis, gray-level variance, zone percentage, zone entropy); Neighboring Gray Tone Difference Matrix (NGTDM)(Coarseness, contrast, busyness, complexity, strength); Gray-Level Dependence Matrix (GLDM)(Dependence entropy, dependence variance, low gray-level emphasis, high gray-level emphasis); and Wavelet-Transformed Features which enhance the ability to detect subtle patterns not evident in the original domain.

### Data handling and preparation for analysis

Features with missing values due to computational errors were excluded. Reproducibility was tested by repetition of the feature extraction on a random sample of patients to verify the consistency of results.

### Feature selection and processing

The extracted radiomic features underwent preprocessing to reduce dimensionality and improve model interpretability. First, highly correlated features (Pearson correlation coefficient > 0.90) were removed to minimize redundancy. Second, features were selected performing Recursive Feature Elimination with Cross-Validation (RFECV) with an Extreme Gradient Boosting (XGB) classifier as the estimator, a tenfold cross-validation strategy and scored by F1. Finally, selected features were standardized using z-score normalization.

### Data splitting

The dataset was randomly divided into three subsets: training (70%), validation (10%), and test (20%). Stratified splitting was applied to preserve class balance in the outcome variable.

### Machine learning model training

Nine ML algorithms were trained to predict one-year edema resolution: Logistic Regression (LR), Naive Bayes (NB), Random Forest (RF), Gradient Boosting (GB), XGB, Histogram Gradient Boosting (HGB), Decision Tree (DT), Support Vector Machine (SVM) and K-Nearest Neighbors (KNN). Model performance was assessed on validation and test datasets with the following metrics: Accuracy, Recall, Precision, F1-score, and Receiver operating characteristic curve (ROC-AUC). The three best models by F1-score and ROC AUC on the test set were further analyzed for interpretability and robustness.

### Model interpretability

To assess the relevance of selected features and provide plausible interpretation of model’s predictions Shapley Additive Explanations (SHAP) were performed. SHAP is a collaborative game theoretic approach which uses feature-based analysis to explain the output of a given ML model [26]. Shapley values estimates both feature importance (contribution magnitude) and direction (negative or positive) toward the selected class. The Shapley value of a feature represents its mean contribution across all feature combinations of a set, revealing their individual impact on the model’s output [29].

## Results

### Study cohort

The final cohort included 123 patients (64.2% women) with a mean age of 57 years. The dataset was randomly divided into 86 cases for training, 12 for validation and 25 for testing. The mean percentage of T2/FLAIR HI resolution at 1 year was 87.2% (SD:2.77; 95%CI: 81.8–92.6%), and 61% of patients achieved ≥ 80% resolution. Tumor volume was not significantly associated with lower edema resolution. However, edema volume was higher in patients with superior resolution rates (18.1 cm^3^ for resolution < 80% vs. 28.3 cm^3^ for ≥ 80%; Mann–Whitney, *p* = 0.015). Grade 1 meningiomas constituted 65% of the cohort, while grade 2 meningiomas were more prevalent in the group with T2/FLAIR HI resolution ≥ 80% (44%vs.20.8%; χ^2^, *p* = 0.023). Notably, atypical meningiomas represented a significant proportion, comprising 41 cases, 33.3% of the cohort. In 8 cases (6.5%), the pathological report was not available. Meningothelial meningiomas accounted for 22.8% of cases, followed by fibrous (20, 16.3%) and transitional (14, 11.4%) subtypes. Less common variants included secretory (4, 3.3%), angiomatous (2, 1.6%), and psammomatous (2, 1.6%) meningiomas. Metaplastic, microcystic, chordoid, and clear cell subtypes were each observed in less than 1% of cases(1, 0.8%). No significant differences were observed in tumor size, histopathological subtype or patient age between groups (Table 1).

### Model performance

**Fig. 3:**
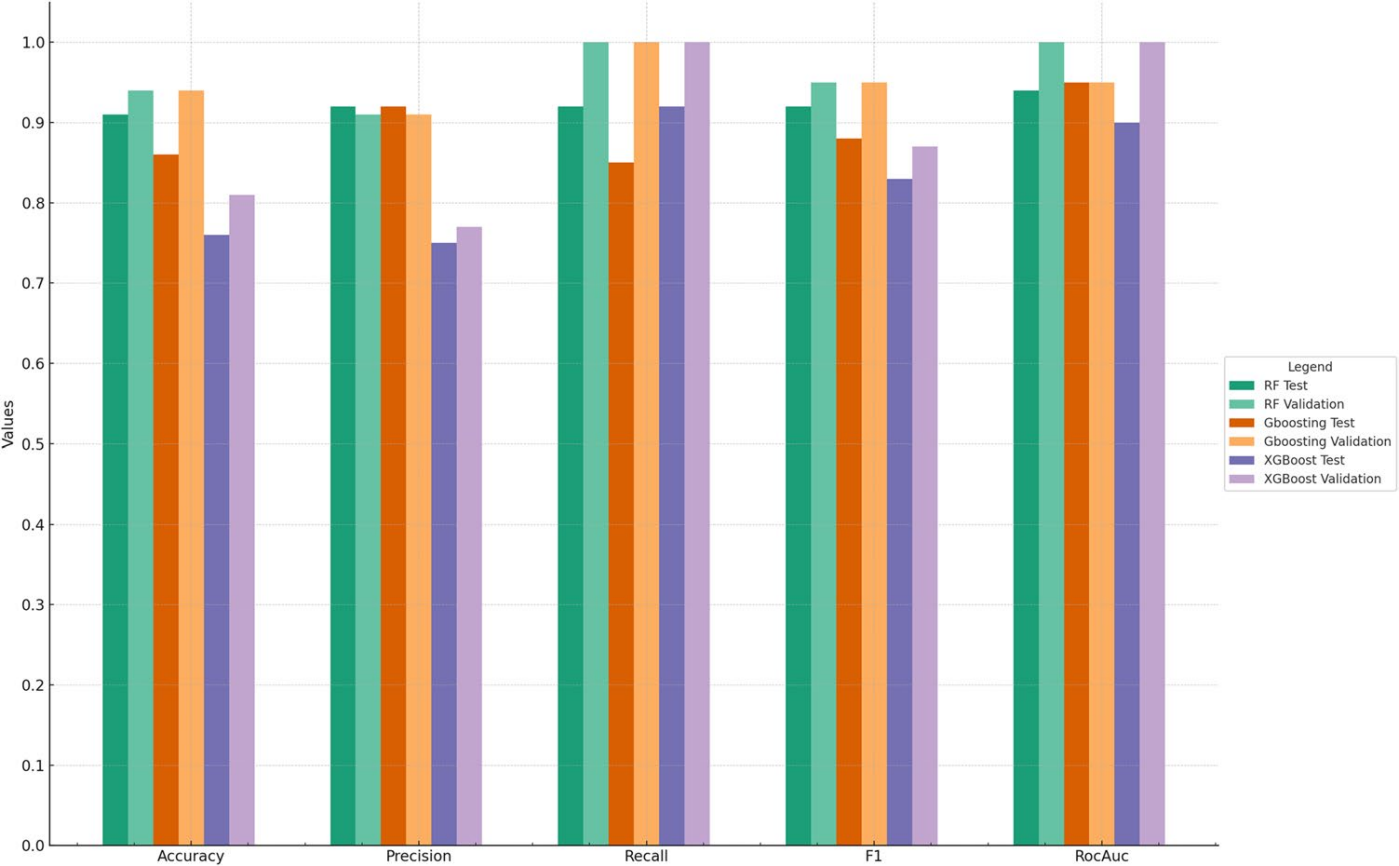
Bar chart summarizing the performance of top 3 models. The darkest and lightest tones of each color refer, respectively, to the results achieved on the test and validation datasets. GBoosting: Gradient Boosting; RF: Random Forest; XGBoost: Extreme Gradient Boosting

Radiomic analysis involved the extraction of 644 radiomic features per patient (322 per label: tumor and edema). After removing correlated features and RFECV, models were trained using 53, 110, 110, 34, 10, and 3 features, corresponding to bW of 5, 10, 15, 20, 25, and 30. Model performance is detailed in Supplementary Table 2. Predictions were most accurate when radiomics were extracted using a bW of 25, and models trained with 10 features. The top-performing models, were RF, GB and XGB (Fig. 3, Table 2). In the test cohort, RF model accurately predicted postoperative T2/FLAIR HI resolution ≥ 80% in over 90% of cases (Accuracy:0.91; Precision:0.92; Recall:0.92; F1-score:0.92; ROC-AUC:0.94) (Supplementary Table 2).

**Table 2:** Performance of top-3 models

Model	BW	Number of Features	Cohort	Accuracy	Precision	Recall	f1	RocAUC
Random Forest	25	10	Validation	0.94	0.91	1.00	0.95	1.00
			Test	0.91	0.92	0.92	0.92	0.94
Gradient Boosting			Validation	0.94	0.91	1.00	0.95	0.95
			Test	0.86	0.92	0.85	0.88	0.95
Extreme Gradient Boosting			Validation	0.81	0.77	1.00	0.87	1.00
			Test	0.76	0.75	0.92	0.83	0.90

*BW* BinWidth, *Roc-AUC* Area Under the curve for Receiver Operating Characteristic

Classification models were further evaluated using learning and calibration curves. These indicated steady improvements in both training and validation accuracy, highlighting the models' generalizability without significant overfitting. The selected models (RF, GB and XGB) demonstrated convergent trends and high validation accuracy with sufficient training samples. Calibration curves further validated the reliability of predicted probabilities, demonstrating alignment between predicted and actual outcomes. This comprehensive evaluation suggests that the chosen models effectively balance classification performance with reliable probability estimations (Supplementary Fig. 1).

### Model interpretability

**Fig. 4:**
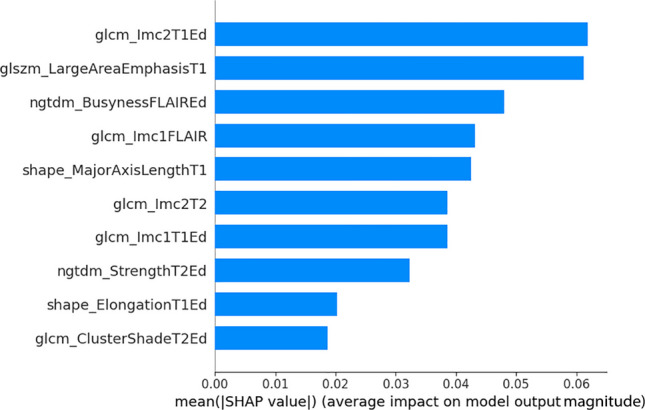
Summary plot of SHAP value for the ten radiomic features utilized by the top 3 performing models. T1, T2 and FLAIR suffixes refers, respectively to radiomic features extracted from the tumor label in the T1 contrast enhanced, T2 and FLAIR sequences. Feature names including the suffix “Ed” correspond to radiomic features extracted from the edema label

SHAP values were computed for all the cases in the test cohort (Table 3). The summary plot (Fig. 4) ranks the most important features in the model and highlights their impact on the output. In the RF model the ten selected features included texture features such as GLCM-LMC2 from the Edema(T1) and Tumor(T2); GLCM-LMC1 from the Edema(T1) and tumor(FLAIR); GLSZM-Large Area Emphasis from the tumor(T1); NGTDM-Busyness from the edema(FLAIR); GLCM-Cluster Shade from the Edema(T2) and NGTDM-Strength from the edema(T2). Shape features such as Major Axis Length from the tumor(T1) and Elongation from the Edema(T1) were also among the top contributors.

**Table 3:** Shap Values for every selected radiomic feature, predicted class and correct classification for the Random Forest Model in the test cohort. (Cases represent original indezes and are not ordered as they were randomly assigned to the test cohort)

Sequence	T1CE					FLAIR		T2						
Label	Tumor		Edema			Tumor	Edema	Tumor	Edema					
Radiomic Feature	Shape MajorAxis Length	GLSZM LargeArea Emphasis	Shape Elongation	GLCM Lmc 1	GLCM Lmc 2	GLCM Lmc 1	NGTDM Busyness	GLCM Lmc 2	GLCM ClusterShade	NGTDM Strength	True Class	Pred Class	Prob Class 1	Correctly Classified
#7	−0.6572	−0.2437	0.1320	0.2410	−0.1700	0.9463	−0.4290	−0.7615	−0.3649	−0.1357	1	1	0.91	True
#12	−1.0693	−0.2314	0.3850	0.5973	−1.4086	0.7420	−0.3488	−1.2484	−0.1157	−0.2301	1	1	0.64	True
#16	0.1441	−0.2353	0.9709	0.0555	0.3315	−0.3341	1.2211	0.8668	−0.0160	−0.2361	1	1	0.77	True
#17	1.0723	−0.2440	−1.9353	−1.3925	1.1985	0.2520	0.6430	−0.6975	0.1162	−0.2077	1	1	0.84	True
#28	0.9948	−0.2420	0.0575	0.6118	−0.5610	−0.1904	−0.3903	0.3345	0.5855	−0.1997	1	1	0.59	True
#45	−0.9661	−0.2028	1.6434	0.0297	0.3766	0.3782	−0.3596	0.7647	0.0585	−0.1304	1	1	0.87	True
#60	−0.6135	−0.2427	0.9252	0.6237	−1.0631	0.1535	−0.5371	−1.5256	0.1122	−0.1493	0	0	0.14	True
#66	0.6411	−0.2099	−0.2325	0.5151	−0.6955	−0.4017	−0.1994	0.0000	0.0000	0.0000	0	0	0.34	True
#71	−1.0567	−0.2451	−0.0157	0.8216	−0.1150	0.8518	−0.5640	0.1407	−0.0018	−0.0412	1	1	0.98	True
#76	1.3268	−0.0243	2.0351	−1.4775	1.8132	−1.7862	0.0069	1.7951	0.1282	−0.2134	1	1	0.78	True
#80	−1.7355	−0.2451	1.2793	−1.7047	1.3592	−0.1878	−0.2119	−0.4271	0.0397	−0.1897	1	1	0.90	True
#91	−0.2194	−0.2165	0.8538	−0.2112	−0.2887	0.5419	0.3269	−0.9748	−0.0105	−0.2380	1	1	0.93	True
#101	0.8886	−0.0340	−1.3077	−0.5693	0.0181	0.6199	−0.6289	0.2642	0.0861	0.4968	1	0	0.45	False
#102	−0.8710	−0.2439	1.9568	0.2645	−1.0079	0.2872	−0.4565	0.7624	−0.1316	−0.2217	1	1	0.84	True
#104	0.3294	−0.2410	1.6784	0.1594	0.9279	−2.1794	2.4843	1.0904	−0.0246	−0.2330	1	1	0.92	True
#119	0.7803	0.1520	−0.5010	−0.0703	−0.2494	−0.7992	−0.4372	0.2586	−0.0734	−0.1827	0	0	0.42	True
#15	−1.2317	−0.2453	0.2857	0.4899	0.7921	0.3006	−0.6155	1.8081	−0.1942	−0.0765	0	0	0.41	True
#22	−0.7653	−0.2443	−1.2452	1.5115	−1.8193	0.6212	−0.6265	−0.9375	0.1241	−0.2042	0	0	0.33	True
#31	−0.2603	−0.2451	−0.6861	1.4836	−2.0677	0.6307	−0.5606	−0.0435	−0.3365	−0.0152	0	0	0.41	True
#48	−0.2590	−0.1970	1.0142	0.3909	−1.5998	0.2237	−0.3457	−1.5867	0.1228	−0.2168	0	0	0.18	True
#51	−0.4586	−0.1184	−0.1407	0.5250	−0.9957	0.7538	−0.4498	−0.2164	0.0867	−0.2103	0	0	0.22	True
#53	−0.7242	1.5170	−1.3716	−2.9452	1.5134	−3.3183	1.4836	1.8637	−0.6044	0.2496	0	1	0.58	False
#54	−0.4035	−0.2441	0.3184	0.7055	−0.0512	1.3660	−0.5589	−1.0691	−0.1094	−0.1169	1	1	0.93	True
#97	−1.6631	−0.2452	0.2315	−0.9181	1.5269	−0.8598	−0.6343	1.4440	6.5022	9.3579	0	0	0.25	True
#100	−0.0036	−0.1717	1.2803	0.3997	−1.1572	−0.0731	−0.3593	−0.9274	−0.0718	−0.2104	1	1	0.65	True

Class 1: Edema Resolution > 80% of the initial Volume; *Pred* Predicted, *Prob* Probability, *T1CE* T1-Contrast Enhanced

**Fig. 5:**
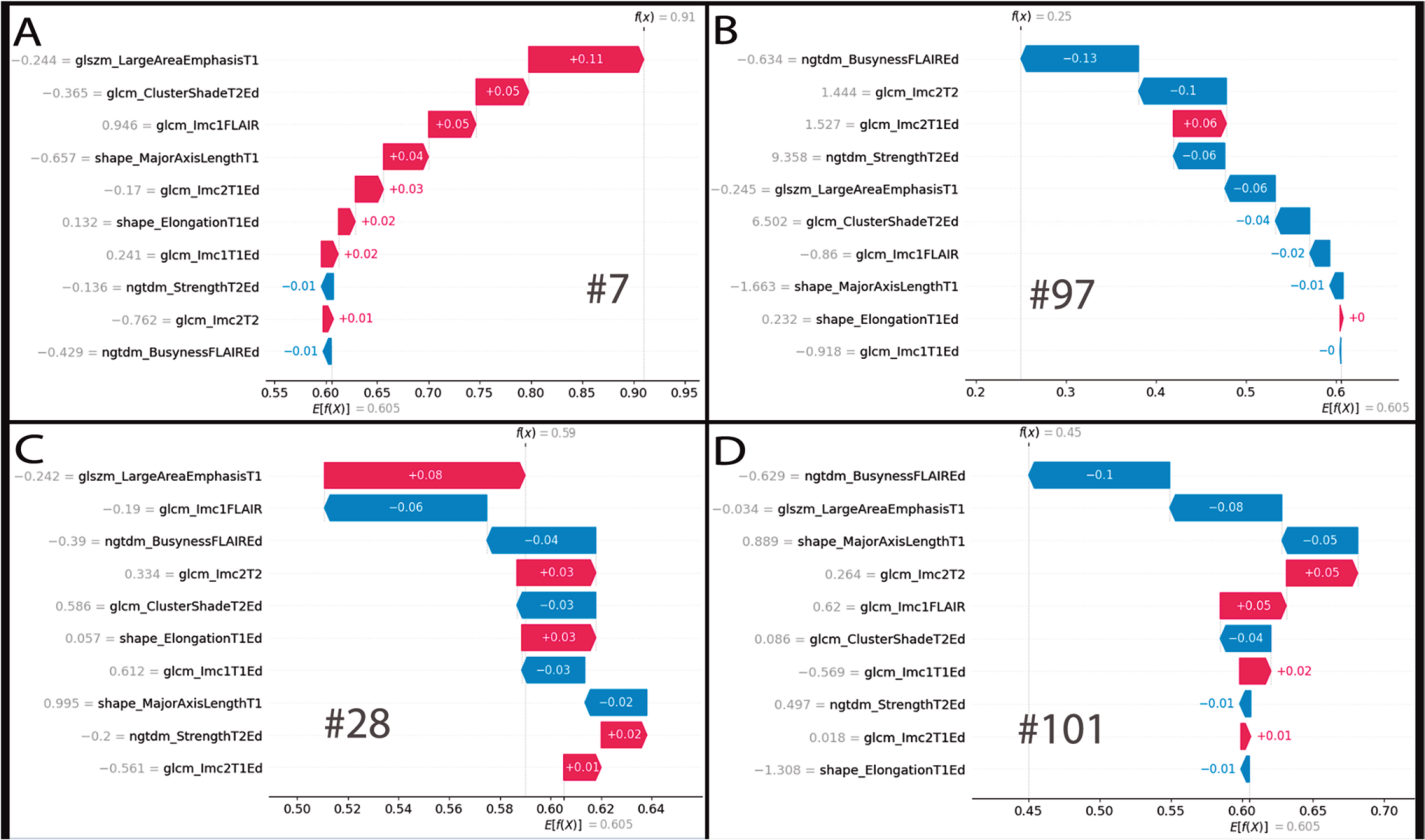
Individual force plot of SHAP for cases 7(**A**), 97(**B**), 28(**C**), and 101(**D**). The individual probability of each case belonging to Class 1 (edema resolution exceeding 80% of the initial volume) is shown under f(x). The numerical and graphical contribution of each radiomic feature is illustrated. Red boxes indicate the influence of a feature in favor of predicting Class 1, while blue boxes indicate its influence against the prediction. SHAP values are displayed on the left of each feature, which are ordered by their impact on the prediction for the respective case. Cases 7 and 97 were assigned to Class 1 and 0, respectively. Case 28 was correctly assigned to Class 1, though the balance of forces was more evenly distributed. Case 101 was incorrectly assigned to Class 0 with a probability of 0.55, a narrow margin that is effectively represented in its force plot

Individual predictions and behavior of each selected radiomic features were calculated (Table 3) and illustrated using force plots (Fig. 5). This allows for case-by-case analysis of model performance and a quantitative understanding of each feature’s contribution to the prediction.

## Discussion

Using features from the tumour and the peritumoral T2/FLAIR HI, the Random Forest model achieved high predictive accuracy for PTBE resolution. Our findings indicate that radiomics-based analysis of preoperative MRI can reveal distinct imaging signatures within PTBE, thereby enabling preoperative identification of regions that represent already established gliosis (which will not resolve after surgery) as opposed to truly reversible edema that disappears postoperatively. While it remains unexplored whether these radiomic patterns represent distinct histopathological entities or are merely reflective of advanced radiological analysis, this study highlights a non-invasive approach for investigating whether meningiomas induce lasting parenchymal changes beyond vasogenic edema, potentially contributing to greater postoperative morbidity [21].

To date, no studies have quantified the proportion of PTBE that constitutes gliosis prior to surgical intervention. A recent systematic review reported rates of persistent PTBE in the literature range from 39 to 83% at final follow-up intervals of up to 157 months, indicating that a substantial proportion of these lesions represents permanent gliotic changes [21]. In the present cohort, the mean resolution of PTBE postoperatively was 87.2, which exceeds values previously reported. This higher rate of edema resolution is likely attributable to methodological differences, specifically the exclusion of patients who developed increased postoperative PTBE.

Previous attempts to predict postoperative PTBE resolution have relied on poorly defined radiological variables that require subjective assessments, such as tumor size, location, heterogeneity and pial blood supply [5, 12]. While not yet validated for this purpose, radiomics offers significant advantages by automating feature extraction and reducing interobserver variability. These advantages add on to their known biological correlation in other brain tumors [1, 6, 15, 28]. While previous radiomic studies focused on tumor-derived features [7, 25], our approach incorporates PTBE radiomics, expanding the feature set while integrating information from the tumor-altered parenchyma. Recent research emphasizes the importance of including PTBE features to predict postoperative progressive PTBE and hemorrhage [14]. In addition, the radiomic fingerprint of PTBE characterizes different brain tumor subtypes and highlights the diversity of brain-tumor interactions and tumor microenvironment [3]. Our approach could lay the foundation for future studies on the radiomic signature and the underlying pathophysiology of persistent postoperative PTBE.

The mechanisms underlying postoperative PTBE evolution remain poorly understood [14, 21]. Hypotheses suggest that factors such as preoperative PTBE extent, tumor grade or subtype, vascular supply, and the preservation of normal venous outflow may play a role in its progression [32]. Surgery itself can exacerbate damage through ischemic, thermal, or mechanical injury to adjacent parenchyma. This is particularly evident in cases where new postoperative T2/FLAIR HI develop. In our cohort, immediate postoperative MRI was not performed in most cases, making it difficult to strictly differentiate between surgery-induced and persistent. Nevertheless, little is known about cases where such damage overlaps with preoperatively identified PTBE regions. Persistent PTBE, i.e. gliosis, or iatrogenic gliotic changes in these areas can lead to long-term morbidity, especially when these HI are situated in eloquent brain regions [5, 32, 34].This issue is not merely theoretical but represents a significant source of postoperative morbidity [32]. Addressing it is crucial, particularly as it could justify, in some clinical scenarios such as refractory seizures, extending surgical resections beyond the enhancing tumor —a concept well-accepted for infiltrative tumors but still contentious for extrinsic lesions [10]. In this context, preoperative, non-invasive assessment of PTBE is critical to differentiate tumor-induced gliosis from transient PTBE.

Recent evidence suggests that specific radiomic features can contribute to unravel the biology, aggressiveness, and recurrence potential of meningiomas [3, 36]. Distinct radiomic features such as high and low intensity large area emphasis, ADC hypointensity, reduced tumor sphericity, and run length non-uniformity can capture tumor heterogeneity and aggressive characteristics to discriminate between low-grade and high-grade [39]. Molecular biomarkers, including Ki-67 proliferation index, NF2 gene status, TERT promoter mutations, and H3K27me3, which are pivotal for tumor classification and prognosis have been the subject of study of recent radiomic studies [9, 17, 31]. This convergence of imaging phenotypes and molecular pathology through radiomics refines risk stratification and supports the development of tailored management plans for meningioma patients. However, the reliability and clinical utility of these radiomic biomarkers depend heavily on standardized imaging protocols, precise segmentation, and consistent application of feature selection and machine learning algorithms [35]. Establishing robust evidence that links radiomic signatures to pathological and molecular findings is essential to harmonize methodologies, improve reproducibility, and ultimately translate these insights into personalized clinical care.

Interpretable models are essential not only for understanding the underlying rationale behind predictions but also for generating new hypotheses and guiding future research. The so-called “black box” issue arises either from the opaque nature of certain ML methods or from the intrinsic complexity of the features they analyze [29]. This understanding is particularly crucial when models are deployed in clinical settings and predictions guide critical decisions.

**Fig. 6:**
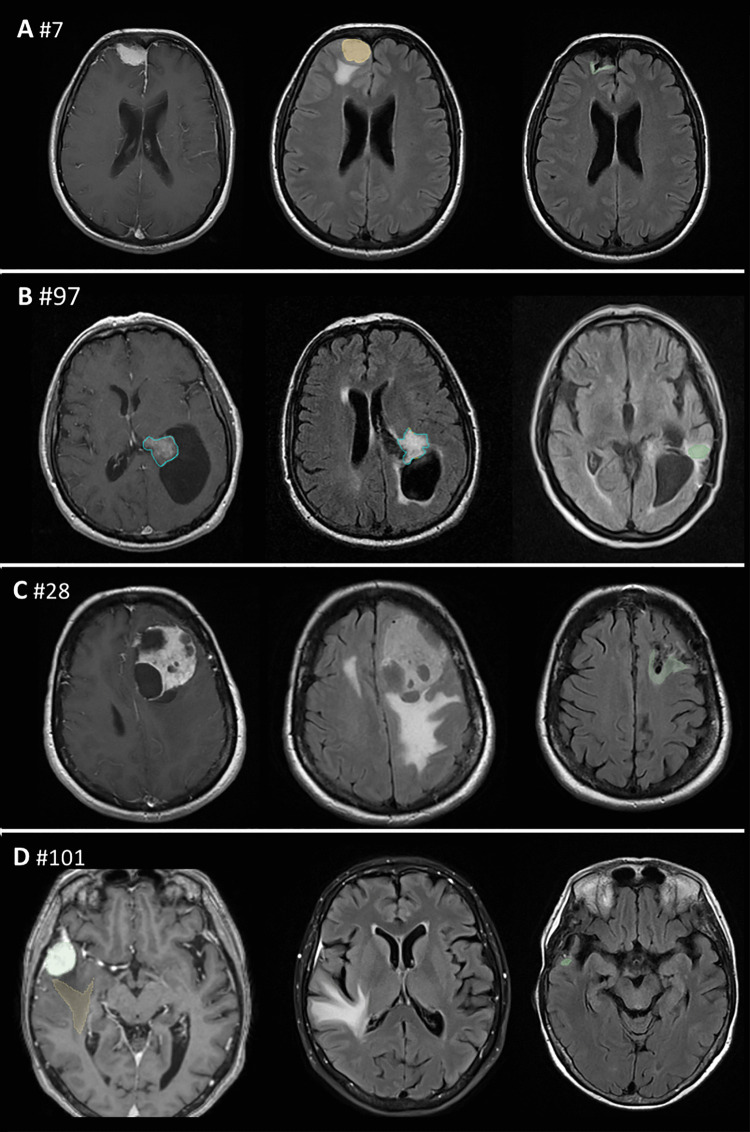
Axial MRI images of four meningioma cases: preoperative T1 contrast-enhanced (left), preoperative FLAIR (center), and postoperative FLAIR (right). Case 7(**A**): Grade II right frontal meningioma with near-complete PTBE resolution after gross total resection. Case 97(**B**): Grade I intraventricular meningioma in an atypical location, showing partial resolution of the periventricular edema and persistence of the edema on the temporal white matter. Case 28(**C**): Left frontal cystic Grade I meningioma with atypical enhancement and > 80% PTBE resolution post-surgery. Case 101(**D**): Tumor with homogeneous enhancement and a typical digitiform HI pattern, with more than 80% resolution postoperatively

In this study, we applied SHAP to enhance the interpretability of our model's predictions. Although, as previously stated, the pathophysiological significance of each radiomic feature remains speculative, a detailed examination of patient imaging alongside these features can provide valuable insights, as illustrated by the following case examples (Figs. 5 and 6):

- Case 7: Despite tumor's atypical nature, the model classified it with high confidence, demonstrating its robustness in handling diverse meningioma subtypes, even when underrepresented in the dataset.
- Case 28: The model assigned it to the high-resolution class with a 0.59 probability, reflecting classification challenges in radiologically atypical cases. The force plot indicated conflicting feature contributions, underscoring the need for improved handling of uncommon radiological patterns.
- Case 97: The model correctly classified the case, with the force plot showing that three of the four strongest contributing features were edema-related. "NGTDM_Busyness" from the FLAIR sequence was the most influential factor. This feature reflects rapid intensity changes which may be associated with morphology and growth dynamics [36].
- Case 101: Despite > 80% edema resolution, this case was misclassified into the low-resolution class (Class 0). The force plot suggested that "axis length" biased the prediction toward Class 0, likely due to training data correlations. Notably, in Case 7, a smaller axis length reinforced high edema resolution, supporting the hypothesis that smaller axis lengths correlate with better resolution outcomes.

SHAP’s interpretability enables precise identification of model weaknesses and subtle biases in the dataset. By highlighting specific areas where the model’s decisions are influenced by imbalanced feature representation, SHAP guides the strategic addition of cases that exhibit alternative feature behaviors. This targeted approach can help balance feature importance and improve the model’s performance, rather than simply increasing sample size, which may not address underlying biases.

## Limitations

This study harbors several limitations that should be acknowledged. First, the relatively small sample size may limit the generalizability of our findings. Second, all cases were operated on in the same department, potentially limiting the external validity of our findings. Additionally, an external validation cohort was not included at this stage due to the novelty of the research, data-sharing constraints between institutions, and the need to build further evidence to support broader recruitment. Nevertheless, cases were referred from a large geographic area, involved MRI scans from different devices, and surgeries were performed by multiple surgeons. Third, while image sequences were co-registered to ensure consistent feature extraction, they were not aligned to a universal 3D space. Although this approach may affect spatially dependent features it was intentionally done to simplify preprocessing to facilitate future modular algorithm development implementing raw DICOM images directly. Fourth, results were sensitive to feature extraction parameters (i.e. bWs), emphasizing the need for standardization and careful interpretation of results. This also suggests that some radiomic features may be particularly dependent on parameter settings, raising concerns about their robustness and reliability across different datasets or workflows. Fifth, the feature selection process, including RFECV and standardization, while necessary, may impact interpretability by excluding potentially meaningful features. Balancing simplicity and model performance with comprehensive feature inclusion remains a methodological challenge. Sixth, the choice of an 80% cut-off for PTBE resolution, while somewhat arbitrary, was made to balance clinical relevance with sample distribution. However, a complete/incomplete resolution threshold may not fully capture clinical relevance and may misclassify borderline cases. Therefore, we opted for a more permissive cut-off as an initial approach that could be further refined in larger or different cohorts. Finally, while SHAP analysis provided valuable insights into model interpretability, its reliability across datasets requires further evaluation to ensure consistency and broader applicability.

Despite these limitations, the study employed robust methodologies to mitigate their impact. Diverse data sources, stratified random splits, and repeated algorithm runs enhanced reliability. Heterogenous sample composition increases external validity while SHAP analysis enabled interpretability. These efforts lay the groundwork for developing a clinically useful, modular, and interpretable machine-learning algorithm for radiomic analysis of meningiomas.

## Conclusion

To date, this is the first study to implement a radiomics approach to predict PTBE resolution. Our findings suggest the existence of distinct biological mechanisms underlying the different evolution of PTBE, warranting further investigation. Given that PTBE significantly contributes to postoperative morbidity, advancing our understanding through non-invasive approaches such as this is essential. Future research should focus on validating these findings in larger cohorts and employing invasive techniques to identify the pathophysiological differences in transient and persistent PTBE.

## Supplementary Information

Below is the link to the electronic supplementary material.

ESM 1 Supplementary Material 1 (DOCX 9.49 MB)

## Data Availability

(Blinded for review) data for secondary use can be obtained through (Blinded for review) (Social and Health Data Permit Authority according to the Secondary Data Act).
