# Supplementary material for: MRI radiomic signature predicts peritumoral brain edema resolution following meningioma surgery
Source: Acta Neurochir (Wien). 2025 Dec 3;167(1):313. doi: 10.1007/s00701-025-06746-7 (PMC12678615; doi:10.1007/s00701-025-06746-7)
Supplement: Supplementary file 1 — Supplementary Material 1 (DOCX 9.49 MB) [file 701_2025_6746_MOESM1_ESM.docx]

**

**

**Supplementary Figure 1:** Learning and Calibration curves for the top 3 models with the best performance.

**
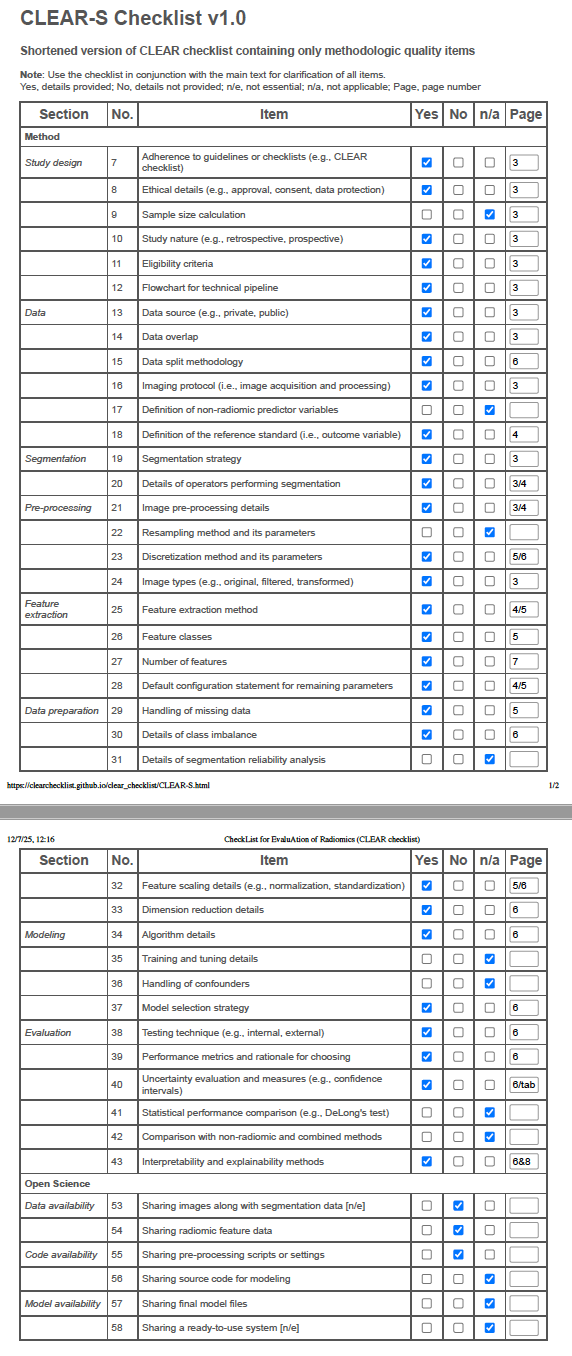
**

**
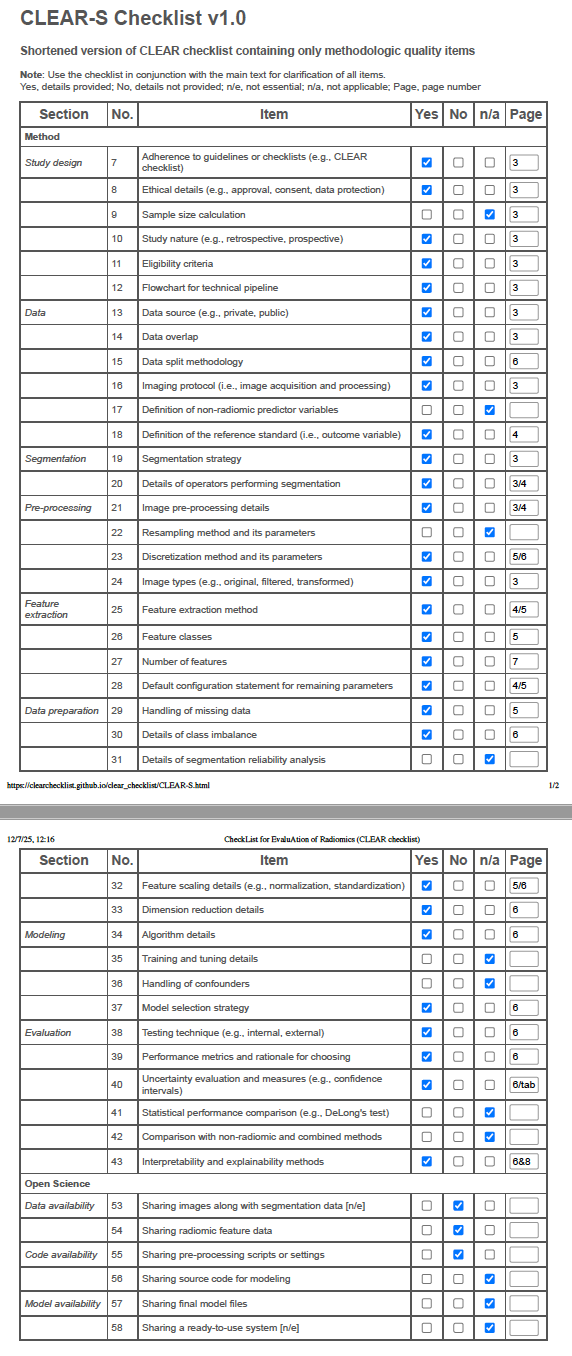
Supplementary Table 1:** CLEAR Checklist

**Supplementary Table 2**: Model Performance.

Acc: Accuracy; BW: BinWidth; DT: Decission Tree; GBoosting: Gradient Boosting; HGB: Histogram Gradient Boosting;kNN: k-Nearest Neighbors; LR: Logistic Regression; Prec: Precision; Rec: Recall; RF: Random Forest; ROC-AUC: Area Under the curve for Receiver Operating Characteristic; SVM: Support Vector Machine ; T: Test; V: Validation; XGB: X Gradient Boosting

| Model | BW | N-Features | V-Acc | V-Prec | V-Rec | V-f1 | V-RocAuc | T-Acc | T-Prec | T-Rec | T-f1 | T-RocAuc |
| --- | --- | --- | --- | --- | --- | --- | --- | --- | --- | --- | --- | --- |
| RF | 25 | 10 | 0.94 | 0.91 | 1.00 | 0.95 | 1.00 | 0.91 | 0.92 | 0.92 | 0.92 | 0.94 |
| Gboosting | 25 | 10 | 0.94 | 0.91 | 1.00 | 0.95 | 0.95 | 0.86 | 0.92 | 0.85 | 0.88 | 0.95 |
| XGBoost | 25 | 10 | 0.81 | 0.77 | 1.00 | 0.87 | 1.00 | 0.76 | 0.75 | 0.92 | 0.83 | 0.90 |
| XGBoost | 20 | 34 | 0.69 | 0.73 | 0.80 | 0.76 | 0.87 | 0.76 | 0.79 | 0.85 | 0.82 | 0.91 |
| HGB | 25 | 10 | 0.81 | 0.77 | 1.00 | 0.87 | 0.98 | 0.71 | 0.71 | 0.92 | 0.80 | 0.91 |
| RF | 5 | 53 | 0.75 | 0.71 | 1.00 | 0.83 | 0.83 | 0.71 | 0.71 | 0.92 | 0.80 | 0.85 |
| Gboosting | 5 | 53 | 0.75 | 0.71 | 1.00 | 0.83 | 0.70 | 0.76 | 0.83 | 0.77 | 0.80 | 0.85 |
| RF | 20 | 34 | 0.88 | 0.83 | 1.00 | 0.91 | 0.96 | 0.71 | 0.77 | 0.77 | 0.77 | 0.88 |
| kNN | 25 | 10 | 0.75 | 0.8 | 0.80 | 0.80 | 0.82 | 0.81 | 0.91 | 0.77 | 0.83 | 0.81 |
| XGBoost | 30 | 3 | 0.63 | 0.7 | 0.70 | 0.70 | 0.70 | 0.81 | 0.91 | 0.77 | 0.83 | 0.81 |
| HGB | 20 | 34 | 0.81 | 0.77 | 1.00 | 0.87 | 0.78 | 0.81 | 0.80 | 0.92 | 0.86 | 0.77 |
| LR | 20 | 34 | 0.88 | 0.90 | 0.90 | 0.90 | 0.85 | 0.76 | 0.79 | 0.85 | 0.82 | 0.80 |
| Gboosting | 20 | 34 | 0.75 | 0.75 | 0.90 | 0.82 | 0.88 | 0.71 | 0.82 | 0.69 | 0.75 | 0.85 |
| RF | 30 | 3 | 0.63 | 0.67 | 0.80 | 0.73 | 0.68 | 0.71 | 0.82 | 0.69 | 0.75 | 0.84 |
| RF | 15 | 110 | 0.69 | 0.69 | 0.90 | 0.78 | 0.93 | 0.71 | 0.77 | 0.77 | 0.77 | 0.81 |
| Bayes | 5 | 53 | 0.75 | 0.75 | 0.90 | 0.82 | 0.65 | 0.76 | 0.83 | 0.77 | 0.80 | 0.77 |
| XGBoost | 5 | 53 | 0.63 | 0.70 | 0.70 | 0.70 | 0.68 | 0.71 | 0.73 | 0.85 | 0.79 | 0.76 |
| RF | 10 | 110 | 0.75 | 0.71429 | 1.00 | 0.83 | 0.86 | 0.67 | 0.69 | 0.85 | 0.76 | 0.78 |
| HGB | 10 | 110 | 0.69 | 0.78 | 0.70 | 0.74 | 0.80 | 0.71 | 0.77 | 0.77 | 0.77 | 0.75 |
| HGB | 15 | 110 | 0.69 | 0.78 | 0.70 | 0.74 | 0.80 | 0.71 | 0.77 | 0.77 | 0.77 | 0.75 |
| LR | 25 | 10 | 0.81 | 0.89 | 0.80 | 0.84 | 0.85 | 0.71 | 0.77 | 0.77 | 0.77 | 0.75 |
| SVM | 25 | 10 | 0.94 | 1 | 0.90 | 0.95 | 0.93 | 0.67 | 0.69 | 0.85 | 0.76 | 0.75 |
| XGBoost | 10 | 110 | 0.8125 | 0.81818 | 0.90 | 0.86 | 0.82 | 0.71 | 0.77 | 0.77 | 0.77 | 0.73 |
| XGBoost | 15 | 110 | 0.82 | 0.82 | 0.90 | 0.86 | 0.82 | 0.71 | 0.77 | 0.77 | 0.77 | 0.73 |
| kNN | 5 | 53 | 0.69 | 0.72 | 0.80 | 0.76 | 0.60 | 0.76 | 0.79 | 0.85 | 0.81 | 0.68 |
| HGB | 5 | 53 | 0.63 | 0.70 | 0.70 | 0.70 | 0.73 | 0.71 | 0.77 | 0.77 | 0.77 | 0.72 |
| Gboosting | 30 | 3 | 0.69 | 0.73 | 0.80 | 0.76 | 0.67 | 0.67 | 0.80 | 0.62 | 0.70 | 0.79 |
| kNN | 20 | 34 | 0.75 | 0.71 | 1.00 | 0.83 | 0.78 | 0.71 | 0.73 | 0.85 | 0.79 | 0.68 |
| DT | 30 | 3 | 0.63 | 0.67 | 0.80 | 0.73 | 0.57 | 0.71 | 0.82 | 0.69 | 0.75 | 0.72 |
| kNN | 30 | 3 | 0.75 | 0.75 | 0.90 | 0.82 | 0.62 | 0.62 | 0.67 | 0.77 | 0.71 | 0.75 |
| LR | 30 | 3 | 0.69 | 0.67 | 1.00 | 0.80 | 0.90 | 0.62 | 0.63 | 0.92 | 0.75 | 0.68 |
| LR | 5 | 53 | 0.75 | 0.75 | 0.90 | 0.82 | 0.76 | 0.67 | 0.75 | 0.69 | 0.72 | 0.70 |
| Naive Bayes | 30 | 3 | 0.75 | 1 | 0.60 | 0.75 | 0.82 | 0.62 | 0.73 | 0.62 | 0.67 | 0.75 |
| LR | 10 | 110 | 0.6875 | 0.85714 | 0.60 | 0.71 | 0.77 | 0.67 | 0.75 | 0.69 | 0.72 | 0.69 |
| LR | 15 | 110 | 0.69 | 0.85 | 0.60 | 0.71 | 0.77 | 0.67 | 0.75 | 0.69 | 0.72 | 0.69 |
| Gboosting | 10 | 110 | 0.75 | 0.80 | 0.80 | 0.80 | 0.85 | 0.67 | 0.75 | 0.69 | 0.72 | 0.68 |
| Gboosting | 15 | 110 | 0.75 | 0.80 | 0.80 | 0.80 | 0.85 | 0.67 | 0.75 | 0.69 | 0.72 | 0.67 |
| DT | 20 | 34 | 0.69 | 0.78 | 0.70 | 0.74 | 0.68 | 0.67 | 0.80 | 0.62 | 0.70 | 0.68 |
| DT | 10 | 110 | 0.50 | 0.60 | 0.60 | 0.60 | 0.47 | 0.67 | 0.71 | 0.77 | 0.74 | 0.63 |
| Naive Bayes | 25 | 10 | 0.44 | 0.67 | 0.20 | 0.31 | 0.72 | 0.62 | 0.86 | 0.46 | 0.60 | 0.75 |
| HGB | 30 | 3 | 0.63 | 0.64 | 0.90 | 0.75 | 0.73 | 0.52 | 0.59 | 0.77 | 0.67 | 0.67 |
| DT | 25 | 10 | 0.88 | 0.9 | 0.90 | 0.90 | 0.87 | 0.62 | 0.73 | 0.62 | 0.67 | 0.62 |
| DT | 15 | 110 | 0.63 | 0.70 | 0.70 | 0.70 | 0.60 | 0.62 | 0.67 | 0.77 | 0.71 | 0.57 |
| kNN | 15 | 110 | 0.63 | 0.63 | 1.00 | 0.77 | 0.50 | 0.52 | 0.62 | 0.62 | 0.62 | 0.63 |
| kNN | 10 | 110 | 0.63 | 0.63 | 1.00 | 0.77 | 0.50 | 0.52 | 0.61 | 0.61 | 0.61 | 0.63 |
| SVM | 30 | 3 | 0.75 | 0.71 | 1.00 | 0.83 | 0.58 | 0.76 | 0.72 | 1.00 | 0.84 | 0.32 |
| Naive Bayes | 10 | 110 | 0.56 | 0.80 | 0.40 | 0.53 | 0.68 | 0.48 | 0.67 | 0.31 | 0.42 | 0.70 |
| Naive Bayes | 15 | 110 | 0.56 | 0.8 | 0.40 | 0.53 | 0.68 | 0.48 | 0.67 | 0.31 | 0.42 | 0.70 |
| DT | 5 | 53 | 0.44 | 0.56 | 0.50 | 0.53 | 0.42 | 0.48 | 0.58 | 0.54 | 0.56 | 0.46 |
| SVM | 10 | 110 | 0.63 | 0.63 | 1.00 | 0.77 | 0.00 | 0.62 | 0.62 | 1.00 | 0.76 | 0.20 |
| SVM | 15 | 110 | 0.63 | 0.63 | 1.00 | 0.77 | 0.00 | 0.62 | 0.62 | 1.00 | 0.76 | 0.20 |
| SVM | 20 | 34 | 0.75 | 0.71 | 1.00 | 0.83 | 0.07 | 0.71 | 0.68 | 1.00 | 0.81 | 0.13 |
| Naive Bayes | 20 | 34 | 0.50 | 0.75 | 0.30 | 0.43 | 0.59 | 0.43 | 0.56 | 0.38 | 0.45 | 0.48 |
| SVM | 5 | 53 | 0.63 | 0.63 | 1.00 | 0.77 | 0.08 | 0.71 | 0.68 | 1.00 | 0.81 | 0.11 |
